# Validation of CD98hc as a Therapeutic Target for a Combination of Radiation and Immunotherapies in Head and Neck Squamous Cell Carcinoma

**DOI:** 10.3390/cancers14071677

**Published:** 2022-03-25

**Authors:** Ayşe Sedef Köseer, Liliana R. Loureiro, Justyna Jureczek, Nicola Mitwasi, Karla Elizabeth González Soto, Julia Aepler, Tabea Bartsch, Anja Feldmann, Leoni A. Kunz-Schughart, Annett Linge, Mechthild Krause, Michael Bachmann, Claudia Arndt, Anna Dubrovska

**Affiliations:** 1 National Center for Tumor Diseases (NCT), Partner Site Dresden: German Cancer Research Center (DKFZ), 69120 Heidelberg, Germany; Faculty of Medicine and University Hospital Carl Gustav Carus, Technische Universität Dresden and Helmholtz-Zentrum Dresden-Rossendorf (HZDR), 01307 Dresden, Germany; aysesedef.koeseer@uniklinikum-dresden.de (A.S.K.); l.loureiro@hzdr.de (L.R.L.); a.feldmann@hzdr.de (A.F.); leoni.kunz-schughart@oncoray.de (L.A.K.-S.); annett.linge@uniklinikum-dresden.de (A.L.); mechthild.krause@uniklinikum-dresden.de (M.K.); m.bachmann@hzdr.de (M.B.); 2 Institute of Radiopharmaceutical Cancer Research, Helmholtz-Zentrum Dresden-Rossendorf (HZDR), 01328 Dresden, Germany; jureczek.justyna@gmail.com (J.J.); n.mitwasi@hzdr.de (N.M.); k.gonzales@hzdr.de (K.E.G.S.); j.aepler@hzdr.de (J.A.); t.bartsch@hzdr.de (T.B.); 3 German Cancer Consortium (DKTK), partner site Dresden and German Cancer Research Center (DKFZ), 69120 Heidelberg, Germany; 4 Tumor Immunology, University Cancer Center (UCC), University Hospital Carl Gustav Carus, Technische Universität Dresden, 01307 Dresden, Germany; 5 OncoRay–National Center for Radiation Research in Oncology, Faculty of Medicine and University Hospital Carl Gustav Carus, Technische Universität Dresden, Helmholtz-Zentrum Dresden-Rossendorf, 01307 Dresden, Germany; 6 Department of Radiotherapy and Radiation Oncology, Faculty of Medicine and University Hospital Carl Gustav Carus, Technische Universität Dresden, 01307 Dresden, Germany; 7 Helmholtz-Zentrum Dresden-Rossendorf, Institute of Radiooncology-OncoRay, 01307 Dresden, Germany; 8 Mildred Scheel Early Career Center, Faculty of Medicine Carl Gustav Carus, Technische Universität Dresden, 01307 Dresden, Germany

**Keywords:** HNSCC, radiotherapy, immunotherapy, CD98hc, SLC3A2, chimeric antigen receptor, radioimmunotherapy, combination therapy, biomarker

## Abstract

**Simple Summary:**

The outcome of patients with locally advanced head and neck squamous cell carcinoma (HNSCC) has not changed for the past decade despite advances in treatment strategies. *SLC3A2*, which encodes CD98hc, was identified as a putative biomarker for HNSCC radioresistance. Chimeric antigen receptor (CAR) T cell therapy presents a promising immunotherapy approach for the treatment of different cancer types. Due to the limitations of this system, including a lack of self-control mechanisms, the adapter UniCAR system with a switchable mechanism was developed. This study demonstrated a synergistic effect of the combination of fractionated irradiation and immune targeting of CD98hc. The sequential combination of fractionated radiotherapy and UniCAR-based immunotherapy revealed itself to be a promising approach for the treatment of high-risk HNSCC patients.

**Abstract:**

Most patients with head and neck squamous cell carcinomas (HNSCC) are diagnosed at a locally advanced stage and show heterogeneous treatment responses. Low *SLC3A2* (solute carrier family 3 member 2) mRNA and protein (CD98hc) expression levels are associated with higher locoregional control in HNSCC patients treated with primary radiochemotherapy or postoperative radiochemotherapy, suggesting that CD98hc could be a target for HNSCC radiosensitization. One of the targeted strategies for tumor radiosensitization is precision immunotherapy, e.g., the use of chimeric antigen receptor (CAR) T cells. This study aimed to define the potential clinical value of new treatment approaches combining conventional radiotherapy with CD98hc-targeted immunotherapy. To address this question, we analyzed the antitumor activity of the combination of fractionated irradiation and switchable universal CAR (UniCAR) system against radioresistant HNSCC cells in 3D culture. CD98hc-redirected UniCAR T cells showed the ability to destroy radioresistant HNSCC spheroids. Also, the infiltration rate of the UniCAR T cells was enhanced in the presence of the CD98hc target module. Furthermore, sequential treatment with fractionated irradiation followed by CD98hc-redirected UniCAR T treatment showed a synergistic effect. Taken together, our obtained data underline the improved antitumor effect of the combination of radiotherapy with CD98hc-targeted immunotherapy. Such a combination presents an attractive approach for the treatment of high-risk HNSCC patients.

## 1. Introduction

Head and neck squamous cell carcinoma (HNSCC) is the sixth most common type of cancer worldwide, with about 890,000 new cases diagnosed annually [1]. The standard of care for patients with locally advanced HNSCC is primary radiochemotherapy (RCT) or, in resectable cases, surgery followed by postoperative radio(chemo)therapy (PORT-C) [2,3]. However, patients may show diverse treatment responses, including treatment resistance, due to HNSCC heterogeneity. In addition to clinical and pathological indicators, biological biomarkers such as hypoxia-associated gene signatures, cancer stem cell marker expression, tumor-infiltrating lymphocytes, and human papillomavirus (HPV) infection are of prognostic relevance in patients with HNSCC treated with radiochemotherapy [2,3,4,5,6]. HPV infection status is widely characterized as a marker of favorable prognosis. In addition, HPV-driven HNSCC shows a better response to radio(chemo)therapy, especially in patients with SCC of the oropharynx [7]. Therefore, additional biological markers for the further stratification of patients with HPV-negative HNSCC and tumor radiosensitization therapies are urgently needed [8]. 

Retrospective biomarker analyses in patients with locally advanced HNSCC treated with primary RCT or PORT-C demonstrated that the expression level of *SLC3A2*/CD98hc is a promising biomarker of both locoregional tumor control (LRC) and distant metastases in patients with HPV-negative tumors, i.e., increased LRC rates correlate with low *SLC3A2* mRNA and protein expression [2,9]. *SLC3A2* encodes CD98hc protein, which forms heterodimeric complexes with light subunits LAT1, LAT2, and xCT to transport branched-chain and aromatic amino acids [10]. CD98hc functions as a chaperon for these light subunits and regulates their stability and plasma membrane localization [11]. Our previous study showed that targeting *SLC3A2*/CD98hc is a promising approach for the radiosensitization of HPV-negative HNSCC. We found that more radioresistant HNSCC cells have high CD98hc expression, and genetic knockdown of CD98hc increases HNSCC radiosensitivity [12]. Accordingly, CD98hc at the protein level might constitute a promising and clinically relevant target for therapeutic intervention in HNSCC.

**Figure 1:**
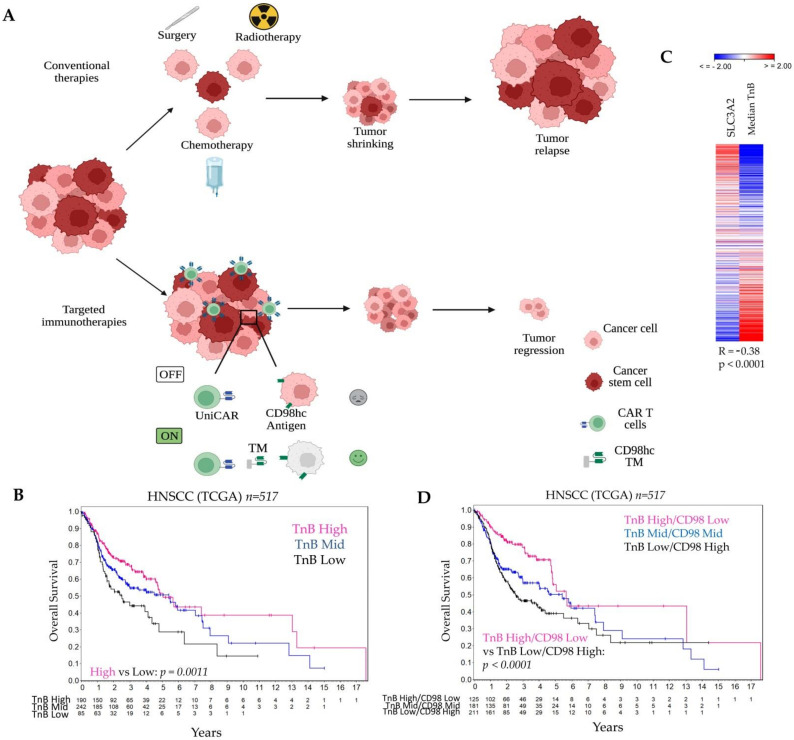
The combination of the CD98hc-targeted immunotherapies and conventional therapies is a promising therapeutic approach for HNSCC. (**A**) Conventional therapies (i.e., surgery, radiotherapy, chemotherapy) reduce tumor mass, but tumor relapse can occur. Targeted immunotherapies with universal chimeric antigen receptors T cells (UniCAR) controllable by targeting modules (TM) can be more efficient with fewer side effects. Created with https://biorender.com, accessed on 8 March 2022. (**B**) Analysis of the TCGA dataset for patients with HNSCC shows an association of the TnB and patient prognosis. (**C**) Expression levels of TnB negatively correlates with *SLC3A2* (CD98hc) gene expression. (**D**) A combination of the low TnB and high CD98hc identifies a high-risk group of HNSCC patients that might benefit from targeted immunotherapies. In Table S1, we provided a list of 84 genes related to T- and B-cell activation and used for the analyses in Figure 1B–D. TM: Targeting Module; TnB: T- and B-cell activation; UniCAR: universal chimeric antigen receptors T cells.

The past decade has witnessed several promising cancer immunotherapy developments, including checkpoint blockade therapy to release the immune system brakes (i.e., PD-1/PD-L1, and CTLA-4) and patient-derived T cells genetically modified to express chimeric antigen receptors (CAR). CARs are equipped with synthetic receptors, which include tumor-binding extracellular domains, transmembrane domains and intracellular domains crucial for T cell stimulation and initiation of target elimination [13,14,15,16]. Numerous clinical trials have demonstrated progress in the efficacy of CAR T therapy in different cancer types [17,18,19,20,21]. However, a limitation of the currently applied conventional CAR T therapy is the lack of self-limiting control mechanisms preventing severe, life-threatening side effects such as cytokine release syndrome [22,23]. A previously developed adapter CAR T platform termed universal CAR (UniCAR) with a switchable mechanism was shown to be controllable by separated short-lived tumor-specific target modules (TM) [24,25,26,27] (see Figure 1A). Compared to conventional CARs, UniCAR T cells do not recognize a surface antigen on tumor cells. Instead, the extracellular binding domain is directed against the 10 amino acid long peptide epitope E5B9 derived from the human nuclear protein La/SS-B [28,29,30]. As naturally occurring La/SS-B is not accessible under physiological conditions for UniCAR binding, UniCAR T cells are switched off and are not able to mediate tumor cell elimination. To turn on the antitumor activity of UniCAR T cells, a separated soluble adaptor molecule, termed TM, is required. This comprises a tumor-specific binding domain (e.g., single-chain fragment variable (scFv), nanobody (nb), small molecules) and the UniCAR epitope E5B9. Thus, TMs function as bridging molecules between tumor and T cells, and are able to activate UniCAR T cells for efficient tumor cell lysis. The UniCAR platform has entered two phase 1 clinical trials (NCT04633148 and NCT04230265). Early clinical results using a CD123-specific TM provide evidence for a well-tolerated, rapidly switchable CAR T product [31]. A recent proof-of-concept study demonstrated the ability of CD98hc-redirected UniCAR T cells to eradicate radioresistant (RR) HNSCC cells in a 2D in vitro model [32]. Additionally, CD98hc-redirected UniCAR T cells could inhibit the growth of RR HNSCC tumors in immunodeficient mice.

The improved survival of HPV-positive HNSCC and the observation that tumor-infiltrating lymphocytes (TILs) are frequently found in these tumors suggest that immune response has an important effect on the prognosis of patients with HNSCC [3,7,33]. Thus, high-risk HNSCC patients with HPV-negative tumors could potentially benefit from novel immunotherapies.

It has become increasingly evident that radiotherapy (RT) can induce immunogenic cell death. Following exposure to ionizing irradiation, tumor cells release damage-associated molecular patterns due to the activation of death-related pathways and the loss of plasma membrane integrity [34]. These molecules are detected by immune cells, resulting in the activation of the adaptive immune system and the release of pro-inflammatory cytokines and chemokines [35,36]. Additionally, several reports have shown that radiation-induced accumulation of cytosolic DNA triggers activation of cyclic GMP-AMP synthase-stimulator of interferon genes (cGAS/STING) pathway, one of the critical mechanisms activating antitumor immune response [37,38,39,40,41]. The immunomodulatory effects of radiotherapy open up an avenue for radioimmunotherapy combination treatment. Indeed, synergistic effects of RT and immunotherapies for systemic antitumor immune responses have been demonstrated in recent years [42,43,44,45,46,47,48,49].

In the present study, we evaluated the ability of the CD98hc redirected-UniCAR T cells to infiltrate and mediate antitumor response in HPV-negative HNSCC 3D models. We also provide evidence for the potential clinical value of the combination of RT with immune targeting of CD98hc based on the UniCAR system.

## 2. Materials and Methods

### 2.1. Cell Culture

This study utilized the established HNSCC cell lines Cal33 (Deutsche Sammlung von Microorganismen und Zellkulturen DSMZ GmbH, Braunschweig, Germany) and FaDu (ATCC, Manassas, VA, USA). The radioresistant (RR) Cal33 derivates were generated as described previously and stably transduced to express red fluorescent protein mCherry [32,50]. Cal33 RR and FaDu cell lines were maintained in DMEM (Sigma-Aldrich, Taufkirchen, Germany) containing 10% FBS (Sigma-Aldrich, Taufkirchen, Germany) and supplemented with 2 mmol/L L-glutamine (Sigma-Aldrich, Taufkirchen, Germany, 10 mmol/L HEPES (Sigma-Aldrich, Taufkirchen, Germany, 1 mmol/L sodium pyruvate (Sigma-Aldrich, Taufkirchen, Germany, 1× MEM nonessential amino acids (Sigma-Aldrich, Taufkirchen, Germany [12]. 3T3 cell lines producing the CD98hc TM or EGFR TM were generated and cultured as previously described [32,51,52]. Before experimentation, the genotypes of HNSCC cell lines were verified using microsatellite polymorphism analyses and confirmed to be *Mycoplasma* negative. All cells were maintained at 37 °C in a humidified atmosphere with 5% CO_2_. 

### 2.2. Purification and Expression of Target Modules (TMs)

As detailed in Arndt et al. [30] and Jureczek et al. [49], CD98hc TM and EGFR TM were constructed by fusing the variable domains of the light (V_L_) and heavy chains (V_H_) of humanized CD98 mAb (MEM-108) or EGFR mAb (Cetuximab) to the UniCAR epitope E5B9. To facilitate the expression of these soluble Ab derivates, TM-producing 3T3 cell lines were generated by lentiviral gene transfer as previously described [32]. UniCAR-TMs were purified via their C-terminal His-tag from cell culture supernatants using Ni-NTA affinity chromatography as described earlier [52]. According to previous studies, the purified TMs were separated via SDS-PAGE, and the concentration and purity were determined via Coomassie Brilliant Blue G250 staining (Sigma-Aldrich, Steinheim, Germany) [52,53,54].

### 2.3. Generation of UniCAR T Cells

The technique for cloning and creating the UniCAR construct has been described in detail elsewhere [55,56]. Briefly, UniCAR is an artificial receptor consisting of (i) an extracellular, humanized scFv derived from the anti-La mAb (5B9), (ii) the hinge, transmembrane and signaling domains of CD28, and (iii) the CD3ζ signaling domains. The E7B6 peptide epitope was incorporated in the extracellular domain, where it serves as tag for detection of UniCAR cell surface expression. The UniCAR construct was further connected via the ribosomal skipping site P2A to the marker protein EGFP allowing independent translation of the UniCAR and EGFP in transduced cells. UniCAR T cell generation was performed as published previously [57]. Briefly, T cells were obtained from buffy coats provided by the German Red Cross (Dresden, Germany) with the informed consent of voluntary donors. The research gained approval by the local ethics committee of the Medical Faculty Carl Gustav Carus, Technische Universität Dresden (EK138042014). Separation of peripheral blood mononuclear cells (PBMCs) was performed with density gradient centrifugation. T cells were isolated with the Pan T Cell Isolation Kit human (Miltenyi Biotec GmbH, Bergisch Gladbach, Germany) [58] and transduced with lentiviruses encoding UniCAR 28/ζ and enhanced green fluorescent protein (EGFP) [57]. According to a previously published protocol [53], T cells were stimulated with T Cell TransAct™ (Miltenyi Biotec GmbH, Bergisch Gladbach, Germany). After 24 h and 48 h, T cells were infected at MOI 2 with viral particles encoding for the UniCAR construct. 24 h after the last transduction, genetically modified T cells were seeded in 24-well G-Rex^®^ plates (WilsonWolf, New Brighton, MN, USA) and expanded for 4–6 days. One day before experiments, transduced T cells were harvested and subsequently cultured in cytokine-deprived medium. By means of the co-expressed enhanced EGFP marker protein, transduction efficiency was calculated on MACSQuant Analyzer 10 (Miltenyi Biotec GmbH, Bergisch Gladbach, Germany).

### 2.4. Spheroid Generation and Culture

Multicellular spheroids were generated in 96-well plates precoated with 1% agarose by seeding 1 × 10^4^ cancer cells in 200 μL to promote spheroid formation [59,60]. For all spheroid experiments, after a formation phase of 2 days, the medium was refreshed by 50% and exchanged every second day (100 μL total/well). Culture medium and incubation conditions were as described under ‘Cell culture’. Spheroid culturing and coculturing with immune cell suspensions in liquid overlay was in principle performed as highlighted earlier with slight modifications [59]. The latter is mainly related to the spheroid initialization period, which could be reduced to the minimum of roughly 48 h for the tumor cells applied in the present study. This protocol allowed producing 500–600 µm spheroids without massive central secondary necrosis. Immune cells were added at a ratio of 5:1 relative to the number of tumor cells in spheroids at the start of the treatment.

### 2.5. Mono- and Combination Treatments

Multicellular spheroids were treated with RT (10 Gy single dose, 2 × 2 Gy or 5 × 2 Gy fractionated RT), chemotherapy (CTx, 0.125 mM cisplatin, Teva GmbH, Ulm, Germany), or radiochemotherapy (RCT, 0.125 mM cisplatin combined with 10 Gy single dose, 2 × 2 Gy or 5 × 2 Gy fractionated RT) (Figure S4A). RCT treatment consisting of 10 Gy single dose, the first fraction of 2 × 2 Gy or 5 × 2 Gy was given 6 h after the start of treatment with cisplatin at a concentration of 0.125 mM. For the UniCAR T cell treatment, spheroids were cocultured with genetically modified UniCAR T cells at a 5:1 effector-to-target cell (E:T) ratio in the presence or absence of CD98hc or EGFR TM for 48 h.

### 2.6. Chromium Release Assay

Tumor cell elimination was determined via standard chromium release assay as described previously [52]. Shortly, 1 × 10^4 51^Chromium (^51^Cr)-labeled Cal33 RR cells were seeded for spheroid formation. After 48 h, they were cocultured with UniCAR T cells with or without TM at an E:T ratio of 5:1 for 48 h and released ^51^Cr was determined in coculture supernatants using a MicroBeta^2^ Microplate Counter (PerkinElmer LAS GmbH, Rodgau, Germany).

### 2.7. Flow Cytometry-Based Killing Assay

Multicellular spheroids were treated with RT, CTx or UniCAR T cell immunotherapy. Forty-eight hours after TM addition, spheroids were washed once with PBS + 2% FBS solution and were trypsinized at 37 °C for 5 min with shaking at 700 rpm. Then, cells were washed with PBS + 0.5% BSA solution, the final volume was adjusted to 100 μL and transferred to a 96-well plate. Six spheroids were used for each measurement. Live and dead cells were distinguished by adding 1 μg/mL of DAPI solution (Sigma-Aldrich, Taufkirchen, Germany). Data was acquired with BD FACS Celesta flow cytometry (BD Bioscience) or MACSQuant VYB Analyzer (Miltenyi Biotec GmbH). All flow cytometric analyses were carried out with FlowJo software (BD Biosciences, Ashland, OR, USA) or MACSQuantify Software (Miltenyi Biotec GmbH, Bergisch-Gladbach, Germany). The percentage of Cal33 RR viable cells was determined using increasing CD98hc TM concentrations in the coculture assays to calculate the half-maximal effective concentration (EC_50_) using nonlinear regression fit in GraphPad Prism 9 [32].

### 2.8. Cytokine Release and Activation Status of UniCAR T Cells

UniCAR T cells were cocultured with or without tumor spheroids in the presence or absence of CD98hc TM in a 96-well plate (E:T = 5:1) in complete RPMI medium with a total volume of 200 µL. After 48 h, cell culture plates were centrifuged for 5 min at 360× *g*, and cell-free supernatant was collected. IFN-γ concentrations were assessed by enzyme-linked immunosorbent assay (ELISA). Human IFN-Gamma ELISA Set and BD OptEIA Reagent Set B were obtained from BD Biosciences, Heidelberg, Germany. Additionally, intracellular staining of granzyme B was performed after 48 h as described previously [55,61,62]. Shortly, 100 μL of cocultured UniCAR T cells were transferred to a new 96-well plate. Extracellular staining, fixation, and permeabilization, as well as intracellular staining, were performed subsequently.

### 2.9. Immunohistochemistry

Multicellular spheroids in 96-well plates were fixed in 4% buffered formalin, dehydrated, embedded in paraffin, and sectioned as 3 μm. Deparaffinization was performed with xylene. Then the sections were rehydrated and microwaved for antigen unmasking. The sections were incubated with CD3 monoclonal antibody (1:50; cat. no. ab828, Abcam, Cambridge, UK) and visualized by standard avidin-biotin-peroxidase complex (Vector Laboratories, Burlingame, CA, USA). Counterstaining was performed with hematoxylin. Infiltrated CD3^+^ T cells were counted blindly by three independent investigators.

### 2.10. TCGA Analysis

For correlation and survival analysis, a dataset of 517 cases for HNSCC from The Human Cancer Genome Atlas (TCGA) was downloaded from cBioportal: http://www.cbioportal.org/, accessed on 21 March 2017. The data were processed following the TCGA policy. Expression heat maps and Kaplan-Meier plots were generated using SUMO software package: http://www.oncoexpress.de, accessed on 4 October 2016. The TnB geneset corresponds to RT2 Profiler PCR Array Human T-Cell and B-Cell Activation https://geneglobe.qiagen.com/us/product-groups/rt2-profiler-pcr-arrays (accessed on 7 May 2020) and includes 84 genes listed in Table S1.

### 2.11. Statistics

Statistical analysis was done by GraphPad Prism 9 (GraphPad Software, San Diego, CA, USA). Paired, nonpaired *t*-test or one-way ANOVA with post hoc Tukey multiple comparison test were used for statistical evaluations. All data were presented as the mean with standard error (SEM). *p* < 0.05 was considered to be statistically significant.

## 3. Results

### 3.1. CD98hc Immunotargeting Eliminates Radioresistant Cells in a 3D In Vitro Model

It is widely accepted that the tumor immune landscape may play an important role in the outcome of HNSCC patients, and can serve as a potential prognostic tool [63,64,65]. Thus, we analyzed immune-related geneset associated with T- and B-cell activation (TnB) and its correlation with the overall survival in 517 HNSCC patients using the TCGA gene expression dataset. For this we calculated the median expression values of quantile normalized TnB geneset and used these values for cutoff scan approach to determine the best expression cutoffs for Kaplan-Meier analysis (Figure S1). We found that patients with low expression of TnB exhibited worse overall survival than those with high gene expression levels (Figure 1B, *p* = 0.0011).

Higher *SLC3A2* gene expression and its association with poor prognosis in HNSCC patients treated with RCT have been reported previously [12]. Hence, we assessed whether the expression levels of TnB correlate with *SLC3A2* gene expression. We found that in TCGA dataset, the median expression levels of the quantile normalized TnB geneset have a moderate negative correlation with *SLC3A2* gene expression (R = −0.38, *p* < 0.0001; Figure 1C).

Notably, the combination of the TnB and *SLC3A2* expression revealed that patients with low TnB and high *SLC3A2* expression showed worse prognosis compared to patients with high TnB and low *SLC3A2* expression (*p* < 0.0001, Figure 1D). This shows that patients with a combination of a low immune infiltrate and high expression of *SLC3A2* are at the highest risk. This supports our study rationale to target CD98hc not only directly but with a cell-based immunotherapy approach. As our previous studies demonstrated that CD98hc is a promising target for tumor radiosensitization in HNSCC, we have used CD98hc-targeted antitumor immunotherapy in combination with experimental RT (Figure 1A).

**Figure 2:**
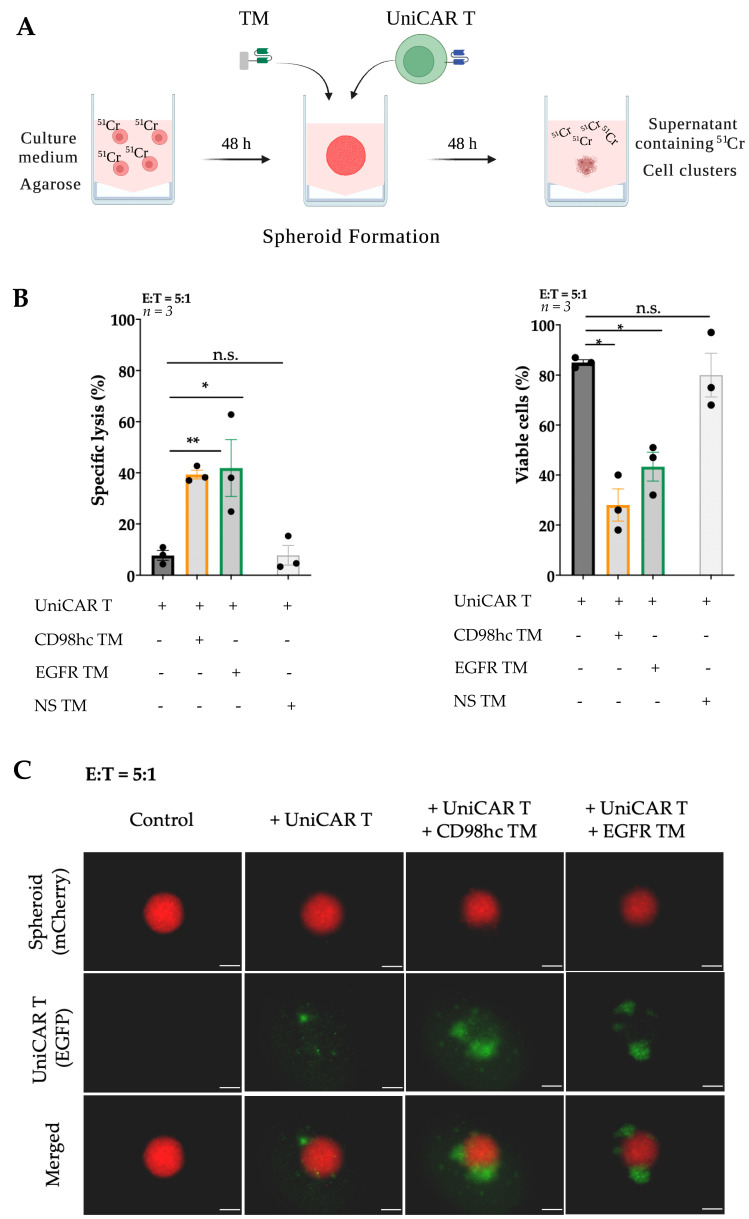
CD98hc-redirected UniCAR T cells destroy Cal33 RR spheroids. (**A**) Scheme of procedures for multicellular spheroid formation, UniCAR T treatment, and standard chromium release assay. Created with https://biorender.com, accessed on 31 January 2022. (**B**) Chromium release assay and flow cytometry analysis showed the efficient elimination of Cal33 RR spheroid cells by UniCAR T cells in the presence of CD98hc TM (50 nM). (**C**) Representative fluorescence images of Cal33 RR spheroids treated with UniCAR T cells in the presence or absence of CD98hc or EGFR TM. Fluorescence images were taken with Axio Observer Z1 (Zeiss, Jena, Germany). Cal33 RR spheroid: red fluorescence, UniCAR T: green fluorescence. Scale bar: 200 µm. Experiments were performed in triplicates. Paired or nonpaired *t*-test were applied to calculate the statistical significance of the treatment efficacy (treated vs. control spheroids); error bars, mean ± SEM. * *p* < 0.05, ** *p* < 0.01. Cr: Chromium; EGFP: Enhanced green fluorescent protein; E:T: Effector-to-target ratio; NS TM: Nonspecific target module; n.s.: not significant.

The potency of the CD98hc-targeted UniCAR T cells to eliminate different HNSCC radioresistant cells in a two-dimensional (2D) model was shown recently [32]. Following this research, we investigated the ability of the CD98hc-redirected UniCAR T cells to eradicate radioresistant HNSCC cells in a three-dimensional (3D) model by 48 h-standard chromium release assays (Figure 2A).

We used previously established [32,50] Cal33 RR cells stably expressing mCherry protein as a model system. Cells were labeled with ^51^Cr and seeded for spheroid formation on 1% agarose-coated plates. Approximately two days after seeding, Cal33 RR formed dense multicellular spheroids. The size of these spheroids was ~500–600 μm (Figure S2A). After formation, spheroids were treated with UniCAR T cells at a 5:1 E:T ratio in the presence or absence of TM targeting CD98hc or nonspecific (NS) TM. After 48 h, CD98hc-redirected UniCAR T cells induced significant cancer cell lysis (Figure 2B, left panel) which was found to be comparable with previously described EGFR TM-redirected UniCAR T cells used as a positive control [32,51]. In the absence of specific TMs or in the presence of NS TM, tumor cells were not significantly affected, showing that targeting of UniCAR T cells was TM-dependent and antigen-specific. These results were validated via flow cytometry by determining the number of viable mCherry-expressing tumor cells (Figure S2B,C and Figure 2B (right panel)). In the presence of CD98hc TM, UniCAR T cells significantly decreased the percentage of viable cells.

Fluorescence imaging showed a noticeable increase of EGFP-expressing UniCAR T cells within 48 h upon their cross-linkage with Cal33 RR cells in 3D culture via the CD98hc TM (Figure 2C), which could potentially indicate the UniCAR T expansion.

### 3.2. CD98hc TM Increases Infiltration of UniCAR T Cells into Tumor Cell Spheroids

**Figure 3:**
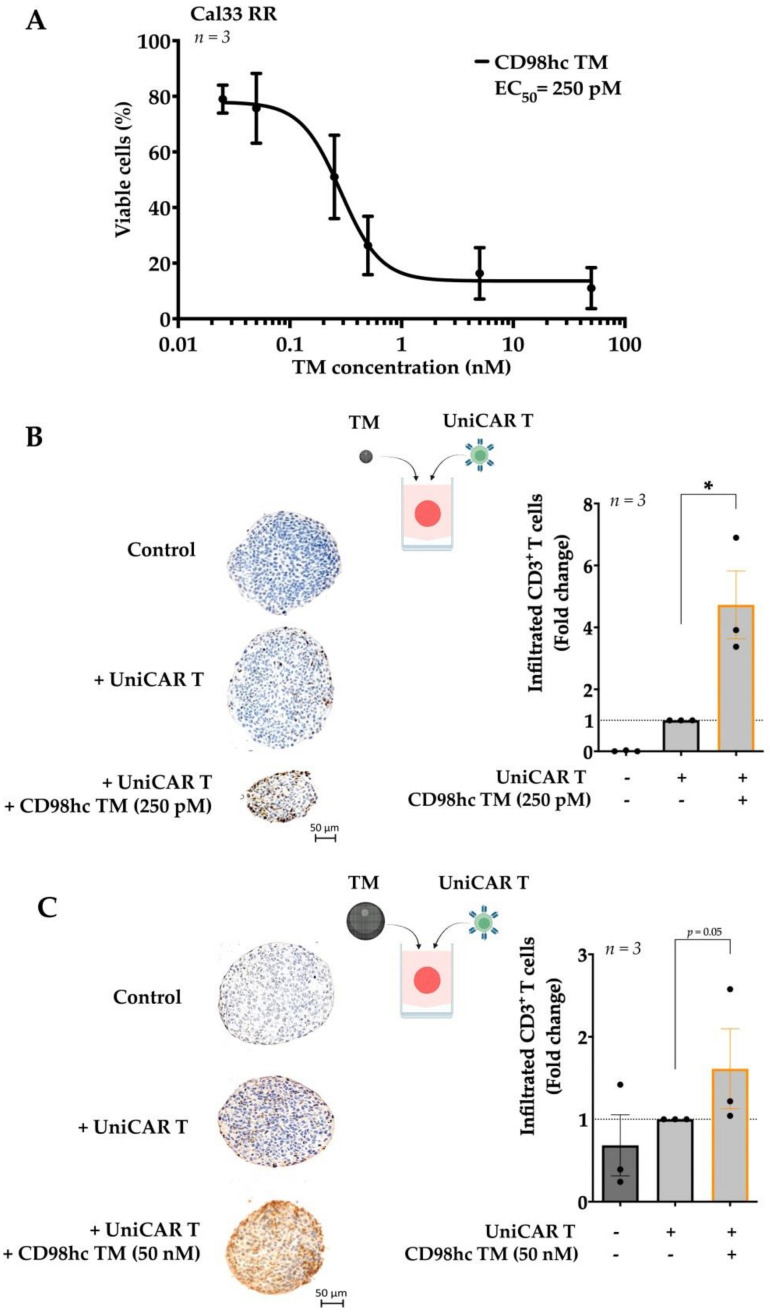
Different CD98hc TM concentrations increase UniCAR T cell infiltration into the tumor spheroids. (**A**) UniCAR T cells were cocultured with Cal33 RR spheroids in the presence of different CD98hc TM concentrations (E:T = 5:1). Half-maximal effective concentration (EC_50_) value was calculated relative to untreated control based on the obtained dose-response curves. (**B**) Immunohistochemical analyses of the median sections of spheroids showed increased infiltration levels of UniCAR T cells in the presence of CD98hc TM at a concentration of 250 pM. Infiltrated CD3^+^ T cells (brown spots) were counted blindly by three independent investigators. (**C**) Immunohistochemical analyses of the median sections of spheroids showing increased infiltration levels of UniCAR T cells in the presence of CD98hc TM at a concentration of 50 nM. Infiltrated CD3^+^ T cells were counted blindly by three independent investigators. Scale bar: 50 µm. Experiments were performed in triplicates. Paired *t*-test was applied to calculate the statistical significance of the infiltration rate (treated vs. control spheroids); error bars, mean ± SEM. * *p* < 0.05.

In a 3D setting, the activity of T cells relies on their infiltration rate, which in the particular case of the UniCAR platform also depends on the concentration of TM. For this reason, we first calculated the half-maximal effective concentration (EC_50_) value of the CD98hc TM in a Cal33 RR 3D model. We found that UniCAR T cells could eliminate tumor cells even at low CD98hc TM concentrations with a half-maximal effective concentration (EC_50_) of 250 pM (Figure 3A).

To investigate the UniCAR T cell infiltration at different CD98hc TM doses, we utilized IHC staining to examine the rate of infiltrated UniCAR T cells in Cal33 RR spheroids. IHC staining demonstrated that EC_50_ concentrations of CD98hc TM already enhanced the infiltration of UniCAR T cells in Cal33 RR spheroids (Figure 3B). Likewise, UniCAR T cells successfully infiltrated the Cal33 RR spheroids at a higher concentration, i.e., 50 nM (Figure 3C). Furthermore, we found the same trend by flow cytometry analysis. This assay evaluated T cell infiltration rate by counting the viable EGFP-expressing UniCAR T cells, however we observed high variability between biological repeats (Figure S3). All in all, our findings suggest that even at lower concentrations of CD98hc TM, UniCAR T cells could infiltrate the Cal33 RR spheroids, resulting in significant elimination of the tumor cells.

### 3.3. Synergistic Antitumor Effect of the Combination of Radiotherapy and Immunotherapy

**Figure 4:**
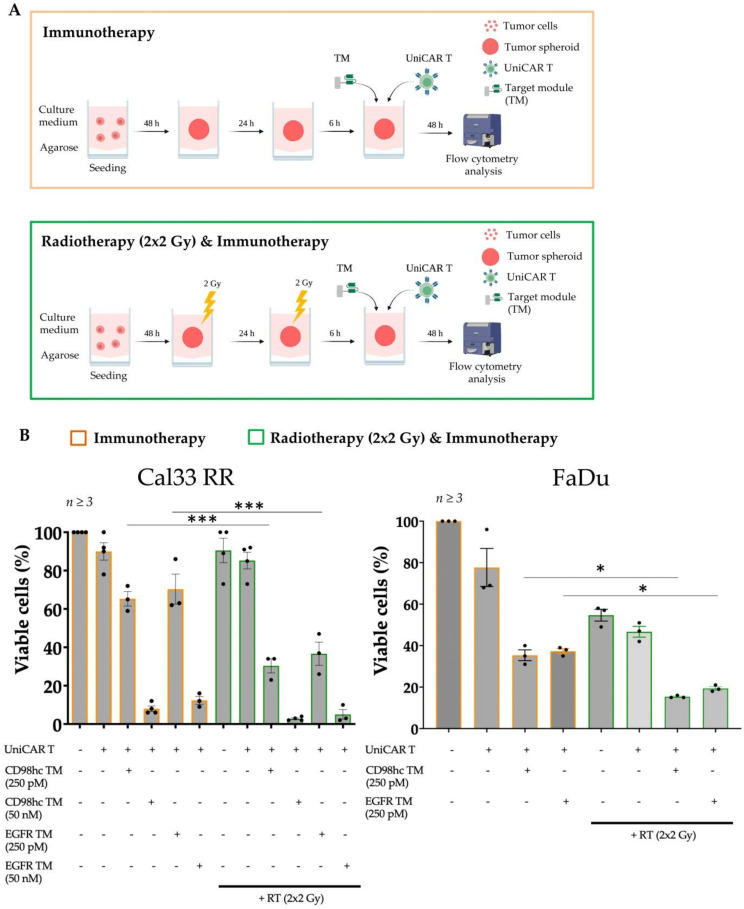
The combination of RT with immunotherapy has a synergistic effect. (**A**) Scheme, and timeline of procedures for combination therapy. Created with https://biorender.com, accessed on 31 January 2022. Upon spheroid formation, spheroids were given 2 × 2 Gy fractionated irradiation in 2 consecutive days. 6 h after final fraction, UniCAR T treatment was performed in the presence or absence of CD98hc TM. 48 h after treatment, viable cell percentages were analyzed by flow cytometry. (**B**) RT in combination with CD98-redirected UniCAR T treatment was significantly more efficient in eliminating Cal33 RR and FaDu spheroids than immunotherapy or RT alone. Experiments were performed in triplicates. One-way ANOVA with post hoc Tukey multiple comparison test was applied to calculate the statistical significance of the treatment efficacy (treated vs. control spheroids); error bars, mean ± SEM. * *p* < 0.05, *** *p* < 0.001. All calculated *p*-values are shown in Figure S5A,B. RT: Radiotherapy.

We investigated the effect of single or combination RT or CTx strategies on tumor cell spheroids and found that RT with a one dose of 10 Gy or with a fractionated dose of 2 × 2 Gy or 5 × 2 Gy, as well as low-dose CTx treatment, did not drastically influence the percentage of viable cells (Figure S4A,B). On the other hand, a combination of single or fractionated RT with low-dose CTx had a noticeable effect. The combination of conventional treatments such as fractionated RT and CTx was toxic to the Cal33 RR spheroids and was sufficient to disintegrate spheroids entirely. Therefore, this strategy was not suitable to test the efficacy of the combination with immunotherapy. Fractionation enhances the impact of radiation on tumor tissue compared to healthy tissue and is the standard treatment technique for HNSCC used in the clinics [66,67]. Therefore, we combined fractionated RT (2 × 2 Gy) and CD98hc-redirected UniCAR T cells to investigate the potential enhancement of the efficacy of conventional therapy like RT in combination with immunotherapy (Figure 4A). Sequential treatment of Cal33 RR spheroids with 2 × 2 Gy fractionated irradiation followed by the treatment with UniCAR T cells in the presence of CD98hc TM using the previously calculated EC_50_ concentrations demonstrated an improved antitumor effect compared to immunotherapy alone (Figure 4B (left panel) and Figure S5A). Similar results were obtained with FaDu cell line that expresses high levels of CD98hc [12], confirming the synergistic therapeutic effect of RT and immunotherapy on different cell lines (Figure 4B (right panel) and Figure S5B).

**Figure 5:**
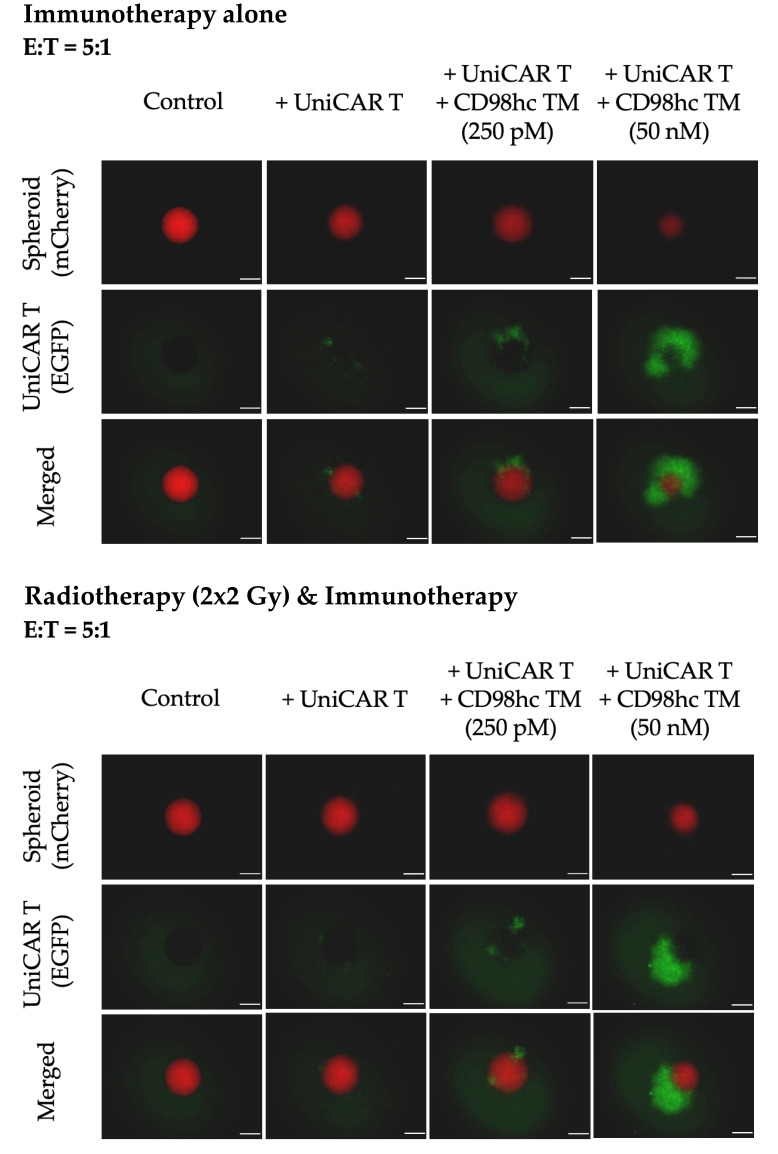
Representative fluorescence images of Cal33 RR spheroids treated with UniCAR T cells in the presence or absence of different CD98hc TM concentrations showing the activation and increase in the EGFP signal of UniCAR T cells in both immunotherapy alone or combination conditions. Fluorescence images were taken with Axio Observer Z1 (Zeiss, Jena, Germany). Cal33 RR spheroid: red fluorescence, UniCAR T: green fluorescence. Scale bar: 200 µm.

Furthermore, fluorescence imaging showed that in the presence of different concentrations of CD98hc TM, an increased EGFP signal was obtained when UniCAR T cells were cocultured either with pre-irradiated or nonirradiated spheroids, which could be potentially attributed to UniCAR T expansion (Figure 5).

**Figure 6:**
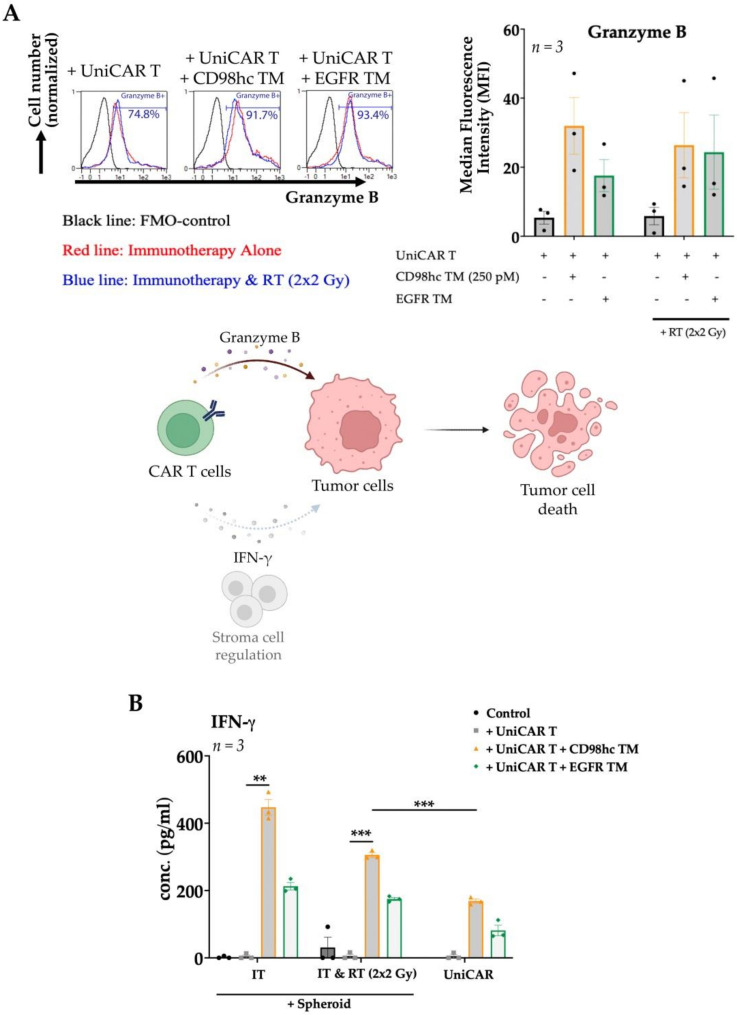
Granzyme B and IFN-γ production of CD98hc-redirected UniCAR T cells. UniCAR T cells were cocultured with Cal33 RR spheroids at an E:T ratio of 5:1 together with CD98hc TM at a concentration of 250 pM. (**A**) After 48 h, UniCAR T cells were analyzed by flow cytometry for intracellular granzyme B expression. Histograms show the UniCAR T cells positive for granzyme B, % (left panel): fluorescence minus one (FMO) control (black line), immunotherapy (red line), RT combined with immunotherapy (blue line). The bar graph shows the median fluorescence intensity (MFI) of granzyme B stained UniCAR T cells (right panel). CAR T cell utilize granzyme B production and can regulate stromal cells via IFN-γ secretion to mediate tumor cell elimination (lower panel). Created with https://biorender.com, accessed on 24 January 2022. (**B**) IFN-γ production profile of UniCAR T cells incubated with or without Cal33 RR spheroids in the presence or absence of 250 pM CD98hc TM (E:T = 5:1). After 48 h, cell-free supernatants were analyzed by ELISA for IFN-γ. IT: Immunotherapy; RT: Radiotherapy; IFN-γ: Interferon-gamma Experiments were performed in triplicates. One-way ANOVA with post hoc Tukey multiple comparison test was applied to calculate the statistical significance (treated vs. control spheroids); error bars, mean ± SEM. ** *p* < 0.01, *** *p* < 0.001.

Aside from analyses of tumor cell viability and expansion of the UniCAR T cells, we next evaluated the upregulation of granzyme B by coculturing UniCAR T cells with Cal33 RR spheroids, fluorescently-labelled antibody-based intracellular staining, and calculating the Median Fluorescence Intensity (MFI). In the presence of CD98hc-specific TM, the intracellular granzyme B expression was upregulated in UniCAR T cells cocultured with either pre-irradiated or nonirradiated spheroids (Figure 6A). We also found that CD98hc-redirected UniCAR T cells specifically secreted IFN-γ when cocultured with Cal33 RR spheroids with or without previous irradiation (Figure 6B). These results suggested that antitumor activity of UniCAR T cell was not affected by irradiation.

## 4. Discussion

HNSCC is one of the most common cancers with a poor patient outcome at locally advanced stages. The tumor immune landscape was shown to influence prognoses. HPV-negative HNSCC has a poor prognosis and possesses an immunologically “cold” landscape with low levels of infiltrating lymphocytes, resulting in a weak antitumor immune response [63]. Thus, these patients could potentially benefit from CAR T-based immunotherapies. In our analysis of the TCGA gene expression cohort, including 517 HNSCC patients, we found that low expression of the gene signature related to T- and B-cell activation is associated with worse prognoses. Previously, Balermpas and colleagues reported that a low rate of CD8^+^ tumor-infiltrating lymphocytes (TIL) was a prognostic parameter for the worse clinical outcome of HNSCC patients treated with PORT-C [33]. Other groups also demonstrated strong peritumoral TIL infiltration in HNSCC patients with lower tumor stage [68,69]. *SLC3A2* has been identified as a promising biomarker for HNSCC radioresistance and a target for tumor radiosensitization [2,9,12,70]. Our findings showed a strong negative correlation between expression of gene signature related to T- and B-cell activation and *SLC3A2*. Therefore, the worse prognosis group of patients with low expression of immune-related gene signature and high CD98hc expression could potentially benefit from a combination of CD98hc-targeted immunotherapies and radiation therapy.

Previous in vitro studies demonstrated that immunotargeting CD98hc using monoclonal antibodies and CAR T cells enhanced immune effector functions against numerous cancer cell lines, including melanoma, ovarian, breast and colorectal cancer cells [71]. However, many tumors might relapse because the persistence of the redirected T cells varies, and tumors can escape by antigen modulation or loss [72,73]. Additionally, the use of CAR T cells is limited by potentially severe, even life-threatening toxicities such as cytokine release syndrome due to a systemic inflammatory response caused by the continuous release of cytokines by CAR T cells [74,75,76,77]. To overcome the limitations of the conventional CAR system and reduce the therapy-induced side effects, a novel CAR T variant termed UniCAR system was developed, whose activity can be regulated in the presence of specific target modules with short half-lives [24,25,26,27]. A recent clinical Phase 1 study assessing the UniCAR system for retargeting AML blasts and using CD123 as a target confirmed that treatment with UniCAR T cells is safe. Moreover, this study demonstrated the switchability of the UniCAR T system [31].

Our previous proof-of-concept study showed that CD98hc-redirected UniCAR T cells exhibit cytotoxicity against highly radioresistant HNSCC cells with elevated CD98hc levels, secreted pro-inflammatory cytokines, and induced perforin and granzyme B production [32]. Additionally, treatment of immunodeficient mice with CD98hc-redirected UniCAR T system resulted in the inhibition of tumor growth. Previous studies have shown that conventional 2D monolayer cell cultures cannot fully recapitulate in vivo intercellular interactions, cell polarity, gene expression or mimic the in vivo tumor microenvironment [78,79,80,81,82]. In this study, we utilized 3D culture systems such as tumor spheroids that provide a potentially superior alternative due to being physiologically more relevant and better replicate the tumor complexities. Differences in the CD98hc-TM redirected UniCAR-mediated tumor cell elimination were evident between 2D and 3D cultures [32]. While radioresistant Cal33 cells exhibited high levels of cell death in 2D, the same cells were much more resistant to UniCAR T cells in the presence of CD98hc TM when they were cultured as 3D spheroids. Nonetheless, we found that UniCAR T cells could expand and subsequently eliminate radioresistant HNSCC 3D spheroids through targeting CD98hc via specific TMs at different concentrations. CD98hc is a well-known marker of immune cell activation that may raise the question of a possible (UniCAR) T cell fratricide upon cross-linkage via the CD98hc TM [83,84,85]. Recently, our group demonstrated that compared to HNSCC cell lines, the density of CD98hc on the UniCAR T cells surface is noticeably lower [32]. Therefore, in the presence of tumor cells, the elimination of CD98hc^high^ tumor cells would occur first and only after that, the lysis of CD98hc^low^ UniCAR T cells would be observed [86]. Low CD98 expression on UniCAR T cells also explains the slight increase of IFN-γ in coculture supernatants upon incubation with the CD98hc TM alone in the absence of tumor cells. However, levels were significantly lower compared to cocultures with Cal33 spheroids. In light of potential clinical application, it is important to note that the lifespans of developed TMs are considerably short. Therefore, the activity of UniCAR T cells and thus side effects like cytokine release syndrome or long-term destruction of healthy tissues (including UniCAR T cells) can easily be regulated and turned “off” by the interruption of TM infusion. We also recognized that the high availability of the CD98hc TM did not inhibit the expansion of the activated UniCAR T cells.

The T lymphocyte infiltration is critically important for immunotherapeutic efficacy in solid tumors. The ability of UniCAR T cells to infiltrate in the 3D spheroid may depend on the specific TM availability in the environment. Our study showed that different doses of CD98hc TM could promote T lymphocyte infiltration into the 3D spheroid and induce significant tumor cell elimination. This observation was also supported by a trend toward the dose-dependent effect of CD98hc TM on T lymphocyte infiltration with our flow cytometry analysis.

Combining conventional therapies, including radiotherapy, with novel precision immunotherapies for cancer management is a recent and highly complementary advance, especially for solid malignancies. Furthermore, in contrast to its successful application for hematological malignancies, the success of CAR T cell therapy in solid tumors is limited so far, and CAR T therapy alone is not sufficient for tumor cure. Our data confirmed that the combination of low-dose chemotherapy with single or fractionated irradiation had a substantial antitumor effect. Recently, Qu and colleagues reported that priming with radiotherapy (40 Gy in 20 fractions) enhanced the efficacy of the CAR T therapy and decreased the toxicity in patients with relapsed/refractory diffuse large B-Cell lymphoma with high tumor burden. This effect was more pronounced than after priming with chemotherapy, suggesting that radiotherapy may be safer and more effective in combination with immunotherapies [87]. Currently, various ongoing clinical trials are testing the effectiveness and safety of a combination of radiation and immunotherapy in different types of cancers [88,89]. For example, DeSelm and colleagues reported that in a pancreatic adenocarcinoma model, low-dose (2 Gy) irradiation increased the sensitivity of tumor cells to CAR T cell killing through TRAIL-mediated death [47]. Therefore, we decided to use 2 × 2 Gy fractionated irradiation with UniCAR T treatment. Our study demonstrated a synergistic effect of the sequential treatment with fractionated irradiation followed by CD98hc-redirected UniCAR T treatment. We also observed a dose-dependent increase of UniCAR T cell expansion in the presence of CD98hc TM leading to the activation of UniCAR T cells for tumor cell elimination. Radiation did not enhance UniCAR T cell elimination of the Cal33 RR spheroids in the absence of CD98hc TM suggesting that this effect is CD98hc TM-specific. Our data also confirmed that UniCAR T-mediated tumor killing was not impaired by irradiation. We also observed a more pronounced effect of radiotherapy on FaDu parental spheroids. Digomann and colleagues recently identified CD98hc as one of the top-scoring proteins upregulated on the membrane of the radioresistant HNSCC sublines. Furthermore, this study also demonstrated the dynamic regulation of CD98hc protein levels after irradiation [12]. Therefore, it is also important to note that the synergistic effect of the combination therapy may be due both to the inherent radiosensitivity of HNSCC cells and to the levels of CD98hc expression on the cell surface, which can be modulated by radiation treatment.

All in all, we show that radioresistant HNSCC cells can be eradicated via CD98hc-redirected UniCAR T system in 3D culture in a highly effective, target-specific, and dose-dependent manner. Our findings also support the concept that multimodal CAR therapy combined with radiotherapy may improve treatment response in solid malignancies.

## 5. Conclusions

In conclusion, we successfully demonstrated that the UniCAR T system could deplete radioresistant HNSCC spheroids by targeting CD98hc. Irradiation followed by UniCAR-based immunotherapy had a synergistic effect and could be a promising approach for the treatment of high-risk HNSCC patients.

## Data Availability

All data supporting the findings of this study and unique biological materials used in this study are available from the corresponding authors upon request.

## Supplementary Materials

The following supporting information can be downloaded at: https://www.mdpi.com/article/10.3390/cancers14071677/s1, Table S1: A list of 84 genes related to T- and B-cell activation and used for the analyses in Figure 1B–D; Figure S1: The cutoff scan approach to determine the best expression cutoffs for Kaplan-Meier analysis using the TCGA gene expression dataset (*n* = 517); Figure S2: Spheroid formation and flow cytometry procedures; Figure S3: Flow cytometric analysis of the UniCAR T cell infiltration; Figure S4: Combination of different treatment strategies; Figure S5: Statistical analysis and *p*-values for comparison between treatment groups in immunotherapy and combination of radiotherapy with immunotherapy.
