# Supplementary material for: Validation of CD98hc as a Therapeutic Target for a Combination of Radiation and Immunotherapies in Head and Neck Squamous Cell Carcinoma
Source: Cancers (Basel). 2022 Mar 25;14(7):1677. doi: 10.3390/cancers14071677 (PMC8997111; doi:10.3390/cancers14071677)
Supplement: Supplementary file 1 [file cancers-14-01677-s001.zip › cancers-1608894-supplementary.pdf]

cancers

MDPI

Supplementary Material: Validation of CD98hc as a Therapeutic Target for a Combination of Radiation and Immunotherapies in Head and Neck Squamous Cell Carcinoma

Ayşe Sedef Köseer, Liliana R. Loureiro, Justyna Jureczek, Nicola Mitwasi, Karla Elizabeth González Soto, Julia Aepler, Tabea Bartsch, Anja Feldmann, Leoni A. Kunz-Schughart, Annett Linge, Mechthild Krause, Michael Bachmann, Claudia Arndt * and Anna Dubrovska *

Table S1. A list of 84 genes related to T- and B-cell activation and used for the analyses in Figure 1CBD.

Gene names

IL12B	*MS4A1*	*IL4*	*CD8B*
IL3	*IL2RA*	*RAG1*	*IL4R*
CD80	*CCL3*	*CD276*	*CD47*
IFNG	*IL7*	*IL18*	*CCR3*
TGFB1	*BLM*	*CD8A*	*MAP3K7*
CD1D	*CXCR4*	*TLR9*	*EGR1*
CSF2	*CD86*	*IL2*	*TLR4*
CD28	*CXCR3*	*RIPK2*	*PTPRC*
IL5	*ADA*	*TLR1*	*IL11*
CD3G	*IL12RB1*	*CXCR5*	*CCR1*
CXCL8	*IL10*	*VAV1*	*TLR6*
IL13	*CCR4*	*IL1B*	*CX3CL1*
FASLG	*IL15*	*TNFSF14*	*TLR2*
CD3E	*CD3D*	*AICDA*	*NCK1*
ICOSLG	*IRF4*	*BCL2*	*MICB*
CD4	*LAG3*	*DPP4*	*IL12RB2*
CD7	*BLNK*	*FAS*	*FOXP3*
SOCS1	*CCR5*	*CD27*	*CD274*
CD81	*CCR2*	*LCK*	*IL18R1*
CD5	*APC*	*CD2*	*CD40*

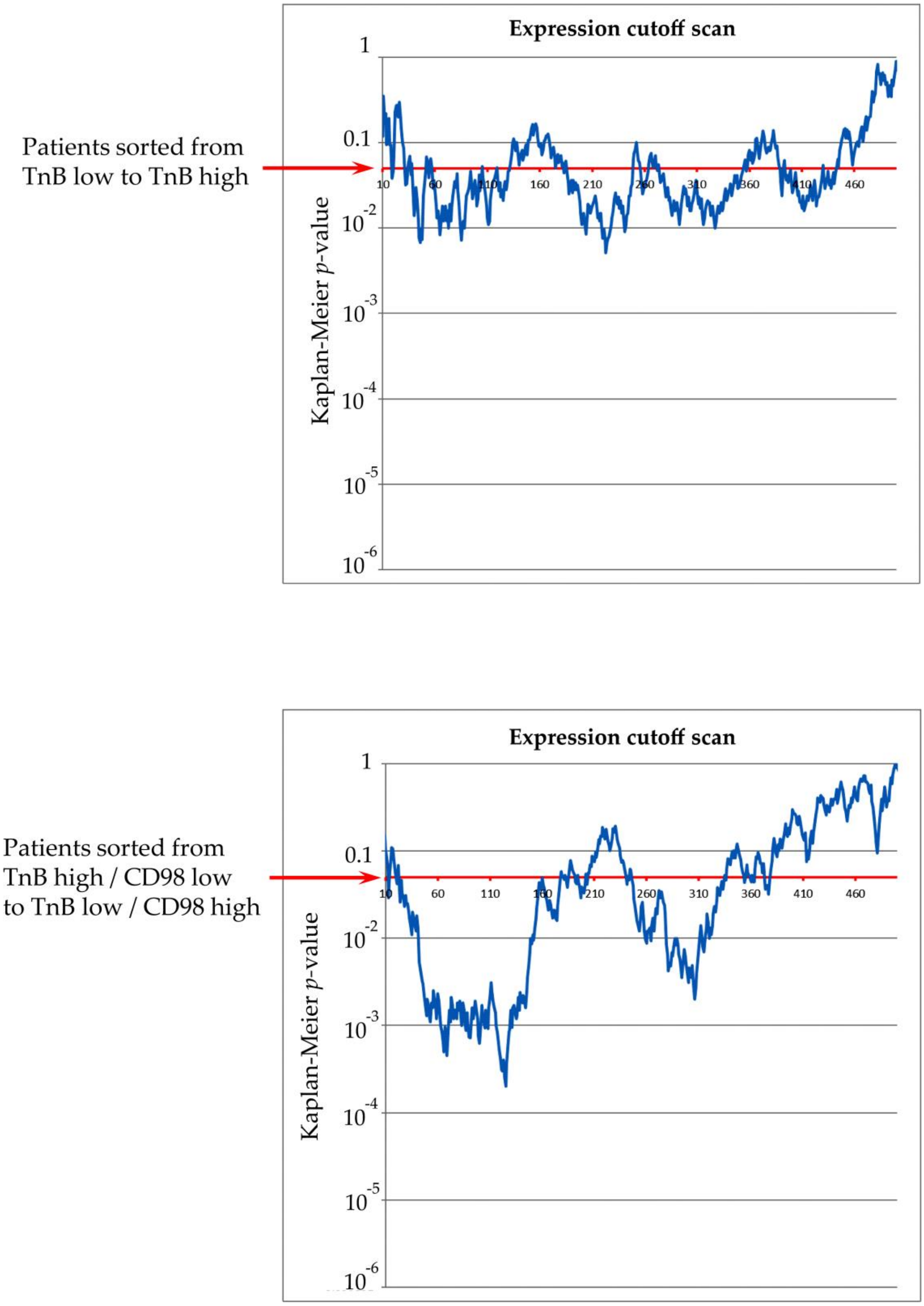

Figure S1. The cutoff scan approach to determine the best expression cutoffs for Kaplan-Meier analysis using the TCGA gene expression dataset (*n* = 517). TnB: T- and B-cell activation geneset.

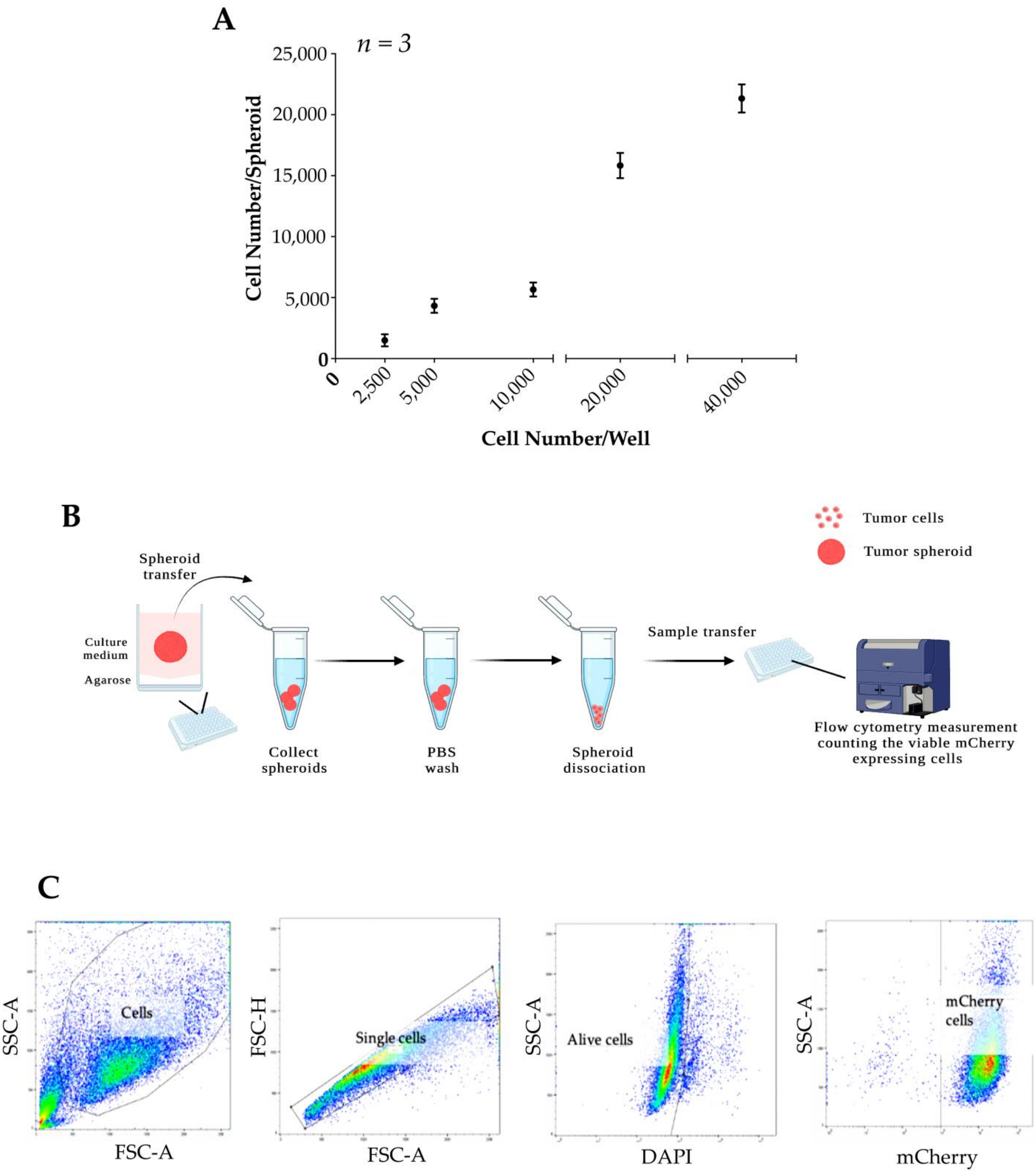

Figure S2. Spheroid formation and flow cytometry procedures. (**A)** Correlation of the number of seeded Cal33 RR cells with spheroid size and cell number/spheroid after 2 days of culturing. (**B)** Scheme of procedures for flow cytometry analysis. Spheroids were collected, washed, and then dissociated. Samples were transferred to a 96-well plate for counting the viable mCherry expressing cells by flow cytometry. (**C)** Flow cytometry gating strategy for detecting and calculating viable mCherry expressing cells.

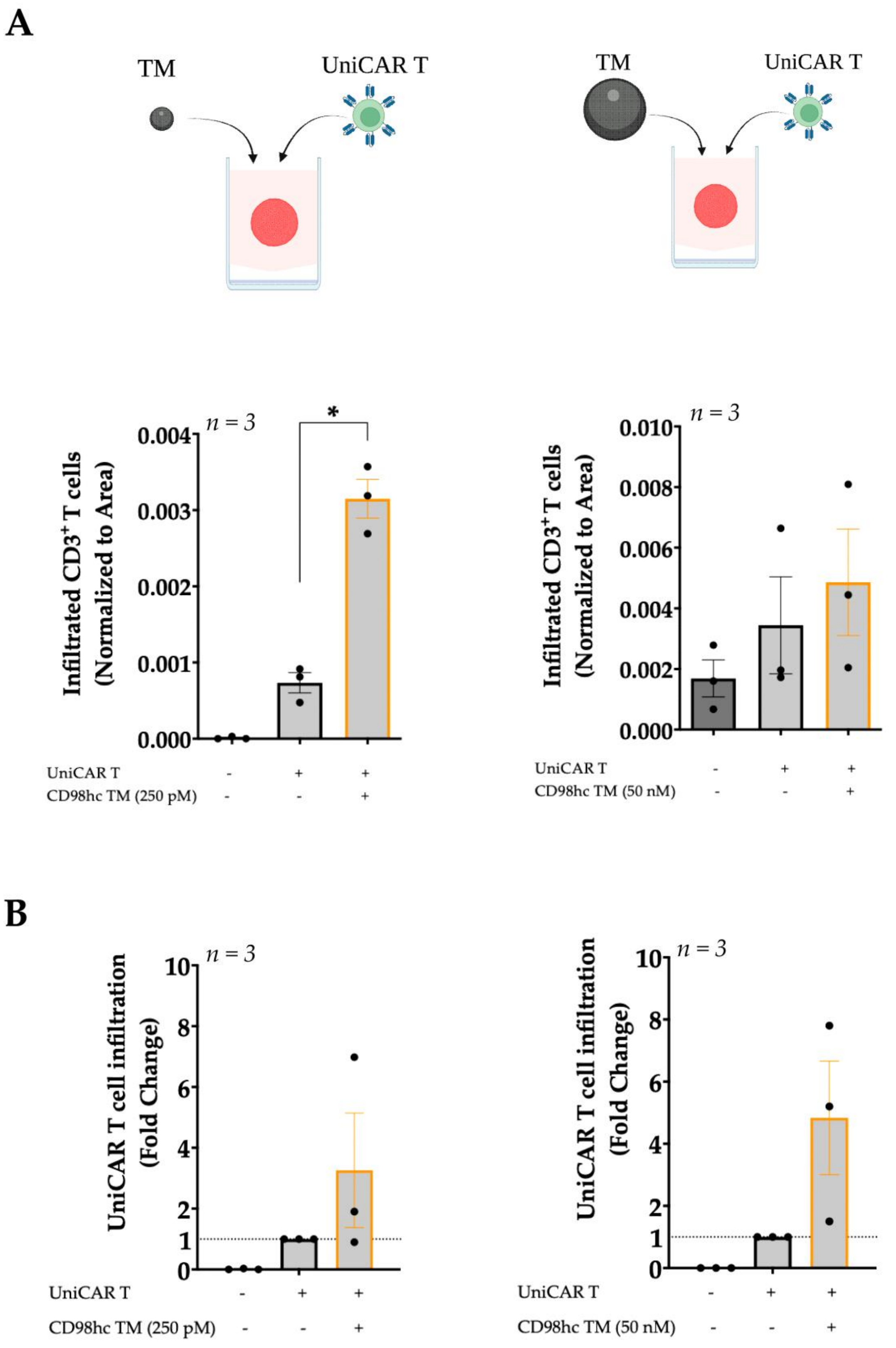

Figure S3. Flow cytometric analysis of the UniCAR T cell infiltration. Cal33 RR spheroids were treated with UniCAR T cells in the presence of lower (250 pM, left panel) and higher (50 nM, right panel) CD98hc TM concentrations. UniCAR T cells were detected based on their EGFP+ signal. Experiments were performed in triplicates. The UniCAR T cell infiltration is shown as the number of UniCAR T in-filtrating cells normalized to the section area (**A**) or as a fold change compared to the experimental condition where only UniCAR T cells were added (**B**). Paired t-test was applied to calculate the statis-tical significance of the infiltration rate (treated vs. control spheroids); error bars, mean ± SEM; * $p < 0.05$.

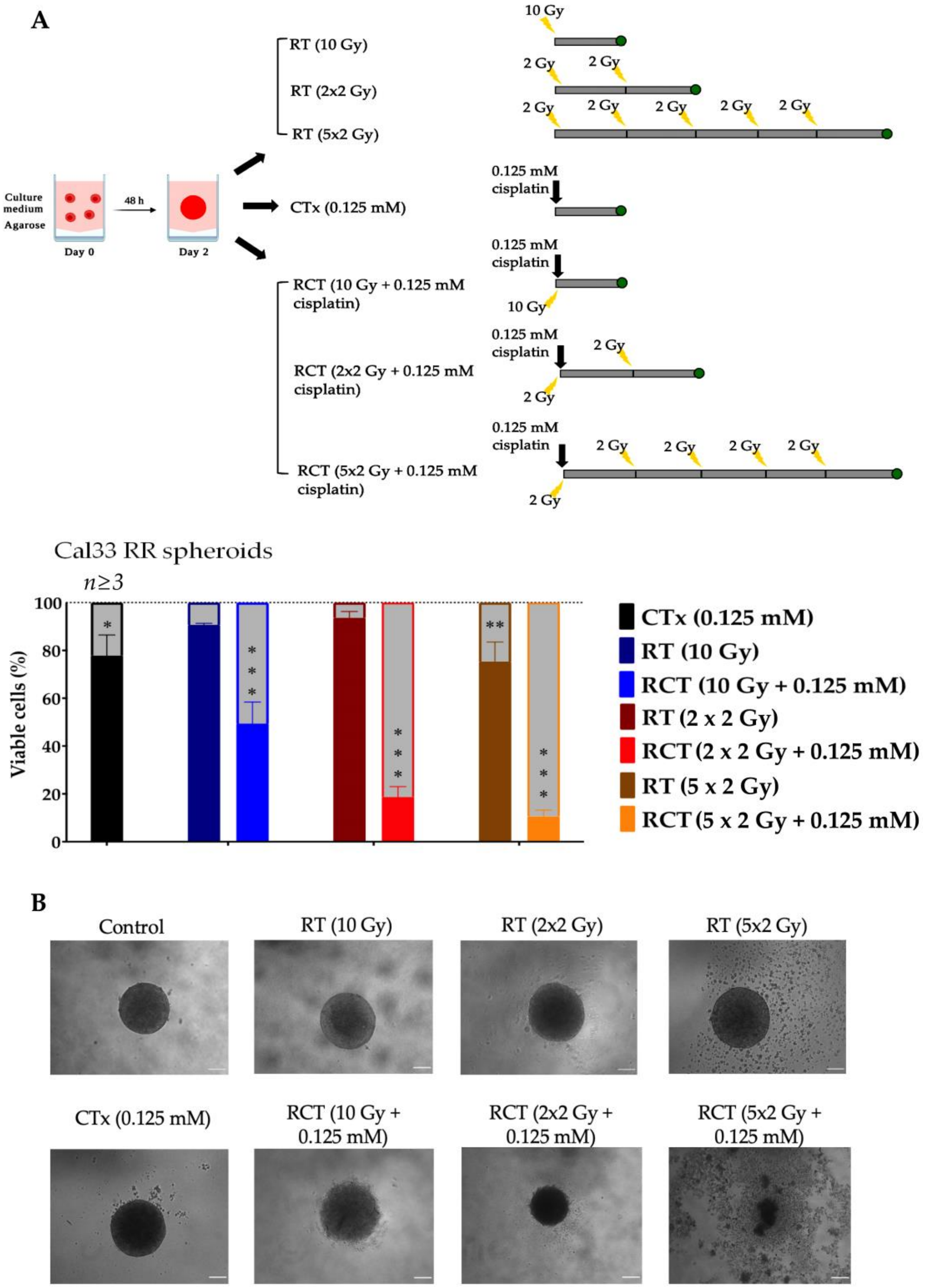

Figure S4. Combination of different treatment strategies. (**A)** Timeline of different treatment strategies including RT (10 Gy, 2x2 Gy or 5x2 Gy), CTx (0.125 mM cisplatin) and RCT (0.125mM cisplatin with 10 Gy, 2x2 Gy or 5x2 Gy). RT and CTx alone did not show a drastic effect. RCT with 0.125 mM cisplatin and 5x2 Gy was the most toxic treatment for Cal33 RR spheroids. RT: radiotherapy, CTx: chemotherapy, RCT: radiochemotherapy. Created with https://biorender.com. Experiments were performed in triplicates. One-way ANOVA with post hoc Tukey multiple comparison tests were applied to calculate the statistical significance of the treatment efficacy (treated vs. control spheroids); error bars, mean ± SEM. * $p < 0.05$, ** $p < 0.01$, *** $p < 0.001$. (**B),** Brightfield images show the spheroid morphology upon different treatment strategies. Scale bar: 200 ◎m.

A

	Control	+ UniCAR T	+ UniCAR T + CD98hc TM (250 pM)	+ UniCAR T + CD98hc TM (50 nM)	+ UniCAR T + EGFR TM (250 pM)	+ UniCAR T + EGFR TM (50 nM)	RT	RT + UniCAR T	RT + UniCAR T + CD98hc TM (250 pM)	RT + UniCAR T + CD98hc TM (50 nM)	RT + UniCAR T + EGFR TM (250 pM)
+ UniCAR T	ns										
+ UniCAR T + CD98hc TM (250 pM)	***	*									
+ UniCAR T + CD98hc TM (50 nM)	***	***	***								
+ UniCAR T + EGFR TM (250 pM)	**	ns	ns	***							
+ UniCAR T + EGFR TM (50 nM)	***	***	***	ns	***						
RT	ns	ns	**	***	ns	***					
RT + UniCAR T	ns	ns	ns	***	ns	***	ns				
RT + UniCAR T + CD98hc TM (250 pM)	***	***	***	*	***	ns	***	***			
RT + UniCAR T + CD98hc TM (50 nM)	***	***	***	ns	***	ns	***	***	**		
RT + UniCAR T + EGFR TM (250 pM)	***	***	**	**	***	*	***	***	ns	***	
RT + UniCAR T + EGFR TM (50 nM)	***	***	***	ns	***	ns	***	***	*	ns	**

B

	Control	+ UniCAR T	+ UniCAR T + CD98hc TM (250 pM)	+ UniCAR T + EGFR TM (250 pM)	RT	RT + UniCAR T	RT + UniCAR T + CD98hc TM (250 pM)
+ UniCAR T	*						
+ UniCAR T + CD98hc TM (250 pM)	***	***					
+ UniCAR T + EGFR TM (250 pM)	***	***	ns				
RT	***	**	*	ns			
RT + UniCAR T	***	***	ns	ns	ns		
RT + UniCAR T + CD98hc TM (250 pM)	***	***	*	*	***	***	
RT + UniCAR T + EGFR TM (250 pM)	***	***	ns	*	***	**	ns

Figure S5. Statistical analysis and *p*-values for comparison between treatment groups in immunotherapy and combination of radiotherapy with immunotherapy. RT: Radiotherapy. One-way ANOVA with post hoc Tukey multiple comparison test was applied to calculate the statistical significance of the treatment efficacy. (**A**) Cal33 RR. (**B**) FaDu. * $p < 0.05$, ** $p < 0.01$, *** $p < 0.001$, n.s.: not significant.
